# Mitigation of Data Packet Loss in Bluetooth Low Energy-Based Wearable Healthcare Ecosystem

**DOI:** 10.3390/bios11100350

**Published:** 2021-09-23

**Authors:** Vishal Varun Tipparaju, Kyle R. Mallires, Di Wang, Francis Tsow, Xiaojun Xian

**Affiliations:** 1 The Biodesign Institute, Arizona State University, Tempe, AZ 85287, USA; vishal.tipparaju@asu.edu (V.V.T.); kyle.mallires@asu.edu (K.R.M.); dwang96@asu.edu (D.W.); tsing.tsow@asu.edu (F.T.); 2 Department of Electrical Engineering and Computer Science, South Dakota State University, Brookings, SD 57007, USA

**Keywords:** bluetooth low energy (BLE), data loss, data transmission, internet of things (IoT), wearable technologies, wireless communication

## Abstract

Bluetooth Low Energy (BLE) plays a critical role in wireless data transmission in wearable technologies. The previous work in this field has mostly focused on optimizing the transmission throughput and power consumption. However, not much work has been reported on a systematic evaluation of the data packet loss of BLE in the wearable healthcare ecosystem, which is essential for reliable and secure data transmission. Considering that diverse wearable devices are used as peripherals and off-the-shelf smartphones (Android, iPhone) or Raspberry Pi with various chipsets and operating systems (OS) as hubs in the wearable ecosystem, there is an urgent need to understand the factors that influence data loss in BLE and develop a mitigation solution to address the data loss issue. In this work, we have systematically evaluated packet losses in Android and iOS based wearable ecosystems and proposed a reduced transmission frequency and data bundling strategy along with queue-based packet transmission protocol to mitigate data packet loss in BLE. The proposed protocol provides flexibility to the peripheral device to work with the host either in real-time mode for timely data transmission or offline mode for accumulated data transmission when there is a request from the host. The test results show that lowered transmission frequency and data bundling reduce the packet losses to less than 1%. The queue-based packet transmission protocol eliminates any remaining packet loss by using re-request routines. The data loss mitigation protocol developed in this research can be widely applied to the BLE-based wearable ecosystem for various applications, such as body sensor networks (BSN), the Internet of Things (IoT), and smart homes.

## 1. Introduction

Bluetooth Low Energy (BLE) is one of the most widely used wireless communication technologies. BLE has ubiquitously diffused into most contemporary electronic devices, such as PCs, smartphones, hubs like Raspberry Pi, smartwatches, fitness trackers, etc. Though the applications of BLE started with indoor proximity detection and positioning using BLE beacons [1,2], the current applications span a wide range of use-cases like multimedia streaming [3], intra-vehicular wireless sensor network for automobiles (IVWSN) [4], smart cities, and so on [5,6].

The healthcare ecosystem is another popular area using BLE technology, due to its high data transmission rate, low power consumption, strong signal strength, miniaturized size, and low cost [7,8,9,10,11,12]. A typical wearable healthcare ecosystem consists of wearable peripherals as the sensor nodes for collecting environmental and health signals and a host/hub unit as the gateway for collecting, processing, integrating, and sharing data. With this architecture, the reliability of the wireless data transmission between the peripherals and the host is essential for the clinical performance of wearable healthcare technology in diagnosis and patient monitoring.

Though BLE technology has been well studied, its practical applications in the wearable ecosystem for reliable and secure data transmission still face challenges, especially in addressing data packet loss. The data packet loss in the wearable healthcare ecosystem is complicated by the following factors. First, a larger packet size is preferred in the wearable healthcare ecosystem. Typical Bluetooth 4.0 only supports 23-byte data packet transmission. However, in the case of Body Sensor Networks (BSN), where multiple wearable devices are involved in the ecosystem, the combined data are streamed with larger packet sizes, up to 255 bytes [13,14]. Second, the BLE chipsets and operating systems (OS) are very diverse in the wearable healthcare ecosystem, especially considering that off-the-shelf smartphones (Android, iPhone) or Raspberry Pi are often used as the hubs [8,15,16]. Third, the optimized factors associated with packet transmission between peripheral wearable devices and central host vary among BLEs from different Original Equipment Manufacturers (OEM). These factors include connection parameters, signal strength, external signal interference, and data corruption [17]. For example, iOS has a set of defined values for connection parameters to the device developers and limits the maximum transmission unit (MTU) size [18]. Android, on the other hand, allows application developers to set the required MTU size. Finally, the interaction between the BLE chipset drivers and the custom application in a host could also cause data packet loss. Most modern operating systems use a Hardware Abstraction Layer (HAL) to abstract internal hardware drivers and parameters from the external application [19,20]. The developer invokes a standard function to request a BLE-specific task and provides a callback handle, which is then invoked back by the OS when the internal drivers complete the requested action. The number of functions exposed by this interface is limited to the most critical operations. It is also possible for the packet to be delivered to the underlying hardware chipset but not passed from the chipset to the requesting application function due to other high-priority tasks running simultaneously on the host. Eventually, the chipset might start dropping packets due to scheduling and priority conflicts. These complex nuances make developing a robust BLE-based data transmission scheme between the wearable peripheral and the host very difficult.

Not only the BLE itself, but also the external environment can cause packet loss. A study shows that the residential microwave oven can interfere the BLE data transmission and cause packet loss in the iOS wearable body area network. However, this study was only conducted on a specific smartphone receiver, which cannot represent the real application scenarios. Wearables are generally independent of the internal workings (i.e., implementation) of master device that connects to synchronize and collect the data [21]. It is also found that the use of multiple connections in a multiple Bluetooth slaves configuration will cause connection instabilities and random packet loss [22]. A compatibility study of the BLE-based platform for electrocardiogram (ECG) monitoring in Wireless Body Area Networks (WBANs) also indicates a significant packet error rate with increasing traffic when the retransmission and flow control mechanisms are not implemented in the BLE sensor to deal with packet loss [23]. Multiple approaches have been proposed to mitigate the data packet loss in BLE, ranging from using orthogonally polarized antennas at the receiver to enabling error correction using redundancy [24]. Recently, Raúl Rondón et al. evaluated three different bounded retransmission schemes of BLE for time-critical communication in the domain of industrial process automation and industrial IoT. A maximum delay below 46 ms and a packet loss rate in the order of 10−5 were achieved, suggesting that BLE could fulfill the requirements of industrial applications [25]. Though these methods help mitigate data packet loss in general BLE use and industrial applications, innovative approaches are still needed specifically for the wearable healthcare ecosystem, where the transmission of critical health information is at stake. In in consumer electronic devices, larger packet sizes are often used, and the BLE chipsets and operating systems (OS) are very diverse. Furthermore, the buffering process and priority handling were overlooked in previous BLE packet loss studies, and thus need to be investigated and considered in the mitigation strategy.

This research attempts to develop a mitigation protocol to address the data packet loss issue in BLE for wearable healthcare ecosystems. Section 2 describes the ecosystem, its components (peripherals and host), and the interaction between the components. Section 3 focuses on evaluating the packet sizes and packet loss in Android, iOS, and Raspberry Pi-based host systems. The various trade-offs among different connection parameters offered by the peripheral devices are investigated in-depth. Finally, a queue-based packet transmission protocol is proposed to mitigate data packet loss in BLE for wearable ecosystems. Section 4 concludes the research and discusses perspectives for future work.

## 2. Materials and Methods (Architecture of the Wearable Healthcare Ecosystem)

The wearable healthcare ecosystem typically monitors two kinds of health information: environmental data such as pollutant exposure, temperature, humidity, barometric pressure, and location; and physiological data such as pulse, SpO2, respiratory rate, body temperature, blood pressure, and biomarkers. Commonly, multiple wearable sensors are used to collect various physiological and environmental data from the subjects to obtain comprehensive information for an accurate analysis of triggers, symptom progression, and feedback on treatment [26,27,28,29]. When dealing with such a wearable healthcare ecosystem, information from multiple sensing units must be collected, merged, synchronized, and indexed to the correct timestamps and user profiles. To achieve this, it is beneficial to have the wearable units comply with a common communication structure that can easily request and integrate relevant information without much hassle. Complying with a common communication protocol will also ensure a straightforward integration of future devices and third-party sensors into the wearable healthcare ecosystem. In this research, we selected a wearable wristband for asthma trigger monitoring and a wearable mask device for respiration monitoring to represent two kinds of health information in the wearable healthcare ecosystem. Another reason for choosing these two wearables is that both need to transmit a large set of environmental or physiological data in their steaming packet, which is important to study the data packet loss behavior in the wearable healthcare ecosystem and to evaluate the performance of the mitigation protocol against data loss.

**Figure 1:**
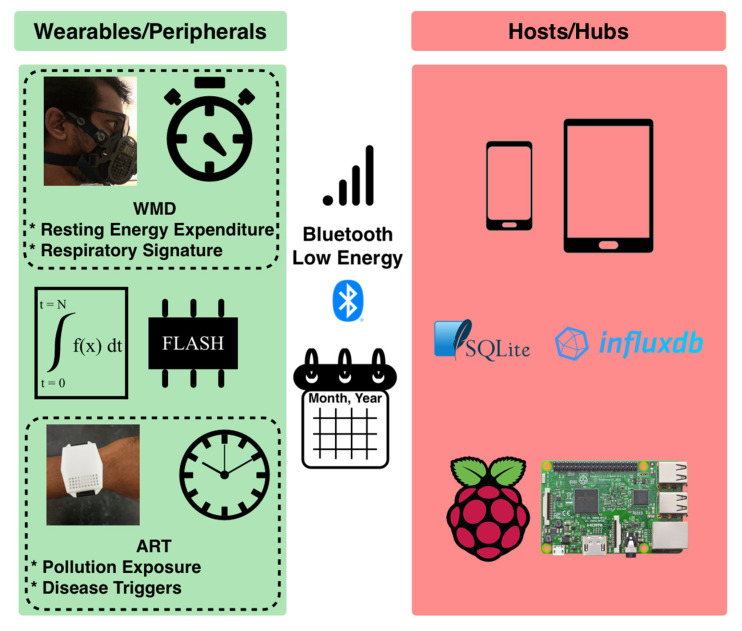
Overview of the components and interactions in a typical BLE-based wearable ecosystem. The peripherals collect and process the necessary signals from wearables and transmit the data to the host over Bluetooth Low Energy.

The components of a typical BLE-based wearable healthcare ecosystem (Figure 1) are defined as follows:

- Wearables (also known as peripherals): These are devices used/worn by the users. They have built-in sensors and microcontrollers to monitor the environment or physiological signals, memory to store data, and a battery to power the devices. The wireless means of data transmission make these devices suitable for tether-free and continuous monitoring in free-living conditions. BLE’s high availability in the mainstream wearables and host devices makes it a suitable choice for wireless transmission in the wearable healthcare ecosystem. Some of the widely used microcontrollers that support BLE and sensor integration include TI CC2640, Nordic nRF51, and nRF52. This ecosystem adopts a piconet style for its network topology, where only one peripheral interacts with the host at any given time.
- Hosts (such as smartphones) and hubs (such as Raspberry Pi): These devices are the intermediate layer between the wearables and the users. The current generation of hosts or hubs are BLE enabled, supporting the communication with the peripherals. These devices also have high data processing capabilities and long battery life. Therefore, they are sometimes used to process the raw data received from the peripherals. Examples include Android Samsung Galaxy phones, iPhones, Android Tablets and iPads. As part of the piconet, the host communicates with only one peripheral at a time. Hosts and hubs use databases to store and retrieve large streams of data on demand. They can also serve as the gateways through which the data can be further uploaded to the IoT cloud, to be accessed by healthcare professionals.

### 2.1. Wearable Devices

In our study, two homemade peripheral devices are used to test the BLE connectivity with the hosts under consideration.

The first device is the “Asthma Research Tool” (ART), built using a TI CC2640 Bluetooth Low Energy wireless MCU. ART is a wrist-worn device that can monitor the user’s personal exposure to various environmental pollutants (Figure 1, bottom left). It has built-in sensors that can monitor ozone, volatile organic compounds (VOCs), temperature, and relative humidity [30,31]. In this application scenario, real-time data transmission is not always required, and the peripheral device does not always need to be within the connectivity range of the host. As shown in Figure 1, ART monitors pollutant exposure throughout the day. Therefore, the device is designed to process sensor signals and store the processed data as packets in the flash memory every minute. The storage sector location is designed to act as a pointer or index to the packet for future retrieval. This design makes the ART less power-consuming since it allows an internal storage of the processed data and on-demand transmission to a host, rather than streaming the data continuously. Whenever the user requests the peripheral’s data using the host, the peripheral retrieves packets from the flash and transmits them to the host over BLE notifications.

The second device is the “Wearable Mask Device” (WMD), built using a TI CC2640 BLE wireless MCU. WMD is a face mask device that monitors the user’s respiration-related physiological parameters based on integrated sensing technologies (Figure 1, top left). The device collects signals from multiple built-in sensors, such as flow sensors, chemical sensors, gyroscope, thermistor, and humidity sensor, at a sampling rate of 40 Hz. The device keeps track of key respiratory parameters such as respiratory rate (RR), tidal volume (TV), respiratory minute volume (VE), and resting energy expenditure (REE) after processing the signals from different sensors. As shown in Figure 1, the respiratory parameters are monitored in real-time. The user is immediately notified of results, such as resting energy expenditure, through the host after information is collected for 11 min [32,33].

### 2.2. Host Devices

**Figure 2:**
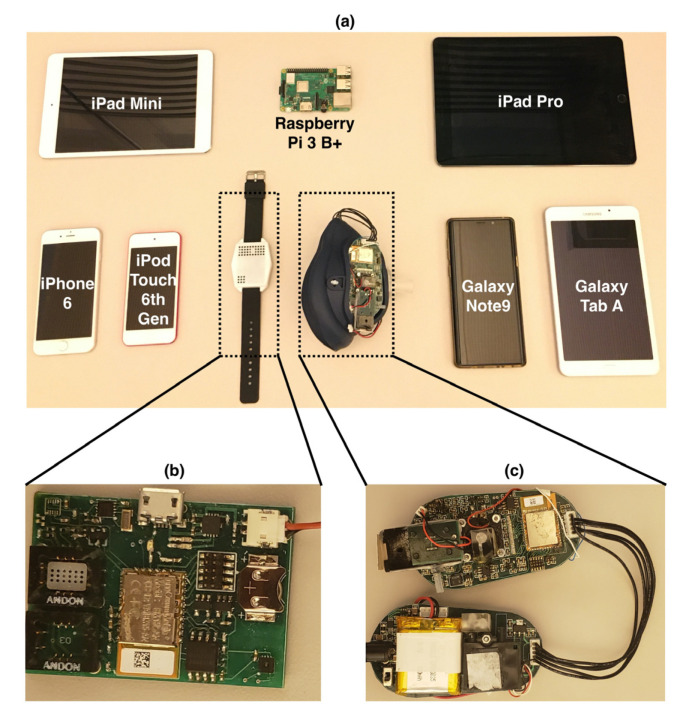
(**a**) Hosts and peripherals used in the BLE data loss evaluation. (**b**) The circuit board of ART, with all the sensors, combined on the top side of the board. (**c**) Paired circuit boards of WMD, which is weight-balanced on both sides of the face mask.

Contemporary smartphones and tablets commonly sold in the market were chosen for the host evaluation study (shown in Figure 2). These devices include:

1. iOS-based devices including iPhone 6, iPad Pro, and iPod.
2. Android-based devices including Samsung Galaxy Note 9 (Android OS 10), and Samsung Tab A 7.0 (Android OS 5).
3. Hubs such as Raspberry Pi 3 B+.

The operating systems and the BLE chipsets of these host and peripheral devices are summarized in Table 1.

### 2.3. Firmware

The firmware for the peripheral devices was developed for TI-RTOS (Real-Time Operating System (RTOS) for Microcontrollers (MCU)) by Texas Instruments (TI) using Code Composer Studio (CC Studio). 

**Figure 3:**
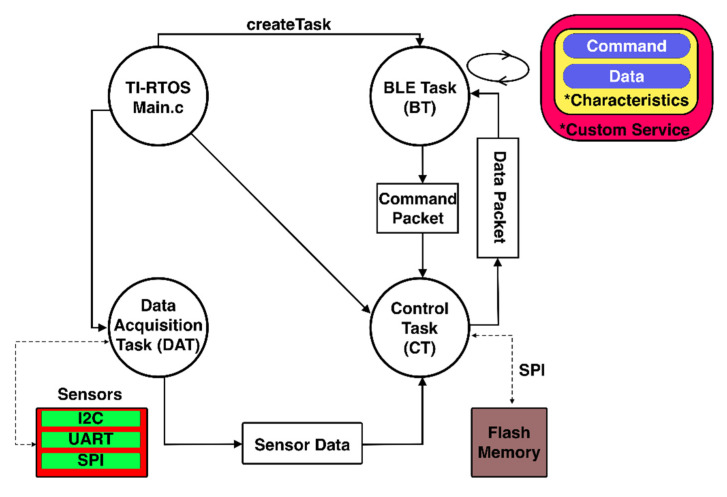
Structure and interaction between tasks in the firmware.

As illustrated in Figure 3, the firmware is designed to have three tasks that have separate functions and responsibilities. In this way, the tasks do not share responsibilities, and failures in one task do not affect the functioning of others.

TI-RTOS provides the BLE-Stack that can start a BLE task (BT) and enable BLE connectivity on CC2640. The BLE task is responsible for advertising the availability of connection, interacting with the BLE-Stack to negotiate the necessary connection parameters requested by the host, and transmitting and receiving data packets sent over by the host application. BT starts one service that contains two characteristics: command and data.

1. For ART, the command is a write-only characteristic that accepts commands from the host application. It forwards the command packet to Control Task (CT) for further action. The data are notification-only characteristics, notifying the host when a new packet is available from CT.
2. For WMD, the command is a write-only characteristic that accepts commands from the host application. It forwards the command packet to the CT for further action. The data are read-only characteristics that maintain the most updated CT packet and are read by the host application.

TI-RTOS also provides functions to create parallel processing tasks, which are used to acquire signals from sensors, store data into flash memory if necessary, and update the BLE task with any data packet requested by the host application. These tasks are divided into two categories.

#### 2.3.1. Data Acquisition Task (DAT)

This task interacts with various built-in sensors integrated on the circuit board via different buses like Inter-Integrated Circuit (I2C), Serial Peripheral Interface (SPI), Analog-to-Digital Converter (ADC), and Universal Asynchronous Receiver/Transmitter (UART). 

For ART, the connected sensors include Temperature and Humidity over I2C, total volatile organic compounds (TVOC) over I2C, real-time clock over I2C, metal oxide semiconductor (MOS) gas sensor over ADC, and Serial NOR Flash Memory over SPI. The sensor data are sampled at 1 Hz, averaged over the time window of one minute. The sensor readings are converted into the final output values if necessary and then passed to the control task.

For WMD, the connected sensors include flow sensor, thermistor, barometric and humidity sensors over ADC, and gyroscope over I2C. It also includes chemical sensors that are tracked using photodiodes; their intensities are measured with the MCU internal ADC. This task samples the sensor data at 40 Hz and implements the breathing tracking algorithm described in [34] to convert, compute, and aggregate parameters such as RR, TV and VE, measurement status, and elapsed time. These values are aggregated cumulatively for each second and passed on to the control task.

#### 2.3.2. Control Task (CT)

This task ensures the synchronization and mediation of data between DAT and BT. It validates the byte array structure of any packets received from BT. It is also responsible for arranging the data from DAT into a correct structure.

For ART, this task controls the conversion of sensor data from DAT into a pre-defined byte array structure for storage in flash memory, the conversion of flash memory data into byte array structure to be transferred to BT, and the erasure of the flash memory when requested by BT. 

For WMD, this task converts the parameters into a pre-defined byte array structure and passes it to BT.

### 2.4. Homemade Applications

Homemade applications have been built using libraries provided by the hosts (XCode, Android SDK, and BlueZ) for peripheral interaction. For Android and iOS applications, the user interacts with the applications to initiate data transmission from the peripheral to the host. For Raspberry Pi, the application scans for the peripheral periodically and collects the data if the peripheral is available. The periodicity of scheduling the application can be configured by the user based on their preferences, e.g., once every hour or twice a day.

We used XCode to build an iOS application [35] and Android SDK for Android application [36]. In the applications, the user can initiate data transfer from the peripheral to the host.

The application for Raspberry Pi was built in BlueZ [37], where the application scans for the peripheral and collects any available data.

**Figure 4:**
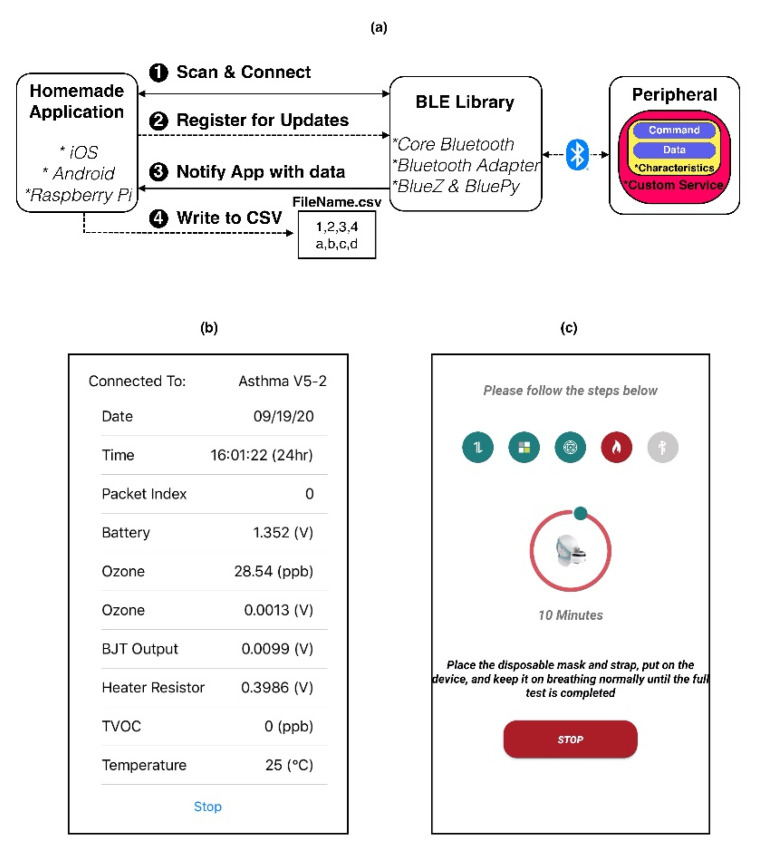
Overview, interaction, and structure of homemade custom applications. (**a**) Design and Interaction between application and system BLE library. (**b**,**c**) The applications built over iOS and Android to collect, process, and store the data.

All the applications follow an observer design pattern [38], where the host application registers with the underlying BLE library for notifications on new data from the peripheral, using a callback function (illustrated in Figure 4). The BLE library is responsible for maintaining the connection and receiving and forwarding the data packet of the peripheral to the registered application. The iOS application uses the “Core Bluetooth” library, provided by the XCode package [39,40]. The Android application uses the BLE service from android.bluetooth.BluetoothAdapter [41]. The Raspberry Pi application uses BlueZ and BluePy services [42,43].

## 3. Results (BLE Performance and Packet Loss)

Since the Connection Intervals, Slave Latency, and Timeouts of the peripheral’s BLE are already specified for iOS, we used these recommended values in the peripheral’s firmware, based on the Accessory Design Guidelines for Apple Devices [18]. To evaluate the reliability of the BLE-based data transmission, we investigated the following parameters that influence the packet loss.

### 3.1. Relationship between Data Loss and Maximum Transmission Unit (MTU) Size

The size of data packets in the BLE Attribute Protocol (ATT) layer is defined by the MTU size requested by the host. We first evaluated the dependency of packet losses on varying MTU sizes and the packet transmission frequency. Each peripheral requires at least 30 bytes to accommodate the necessary sensor data. Therefore, packet sizes in multiples of 30 were chosen for tests. In the tests, the peripheral transmitted a fixed number of packets (1440 packets numbered sequentially) to the host. The host and peripheral transferred packets over BLE using communication events (CE). These events were exchanged at a fixed periodicity, which is described by connection interval (CI) in milliseconds (ms). Based on the design guidelines [18], connection intervals (CI) ranging from 30 ms to 105 ms were considered. As mentioned in previous work [44], the maximum number of packets per CI varies between 4 and 6 for iOS and Android, respectively. Therefore, have tested the packet transmission frequency from 38 Packets/sec (CI = 105 ms, 4 packets per CI) to 200 Packets/sec (CI = 30 ms, 6 packets per CI).

**Figure 5:**
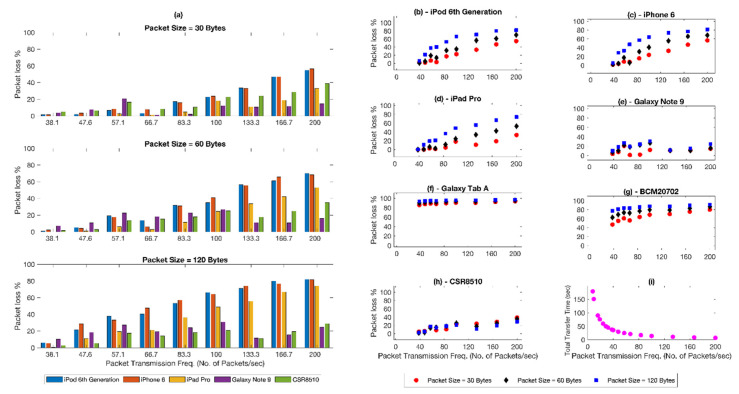
Evaluation of BLE connectivity with hosts over varying connection parameters. (**a**) Influence of packet sizes and transmission frequency over packet loss at the receiver. (**b**–**h**) Packet losses for individual host device under consideration. (**i**) The relation between transmission frequency and total transfer time.

The overall packet losses (as seen in Figure 5a) were proportional to the packet transmission frequency. For a given transmission frequency, an increase in packet size led to an increase in packet loss. While the average trend was mostly the same across various hosts, some showed slightly varying trends (like CSR8510). Figure 5b–h show the relationship between packets loss and packet transmission frequency on different hosts. The test results suggest.

1. The chance of packet loss declines with the decreasing transmission frequency.
2. As shown in Figure 5i, reducing transmission frequency rapidly increases the total transfer time. In high load applications that require higher throughput, the long total transfer time can be mitigated by bundling the data packets.

**Figure 6:**
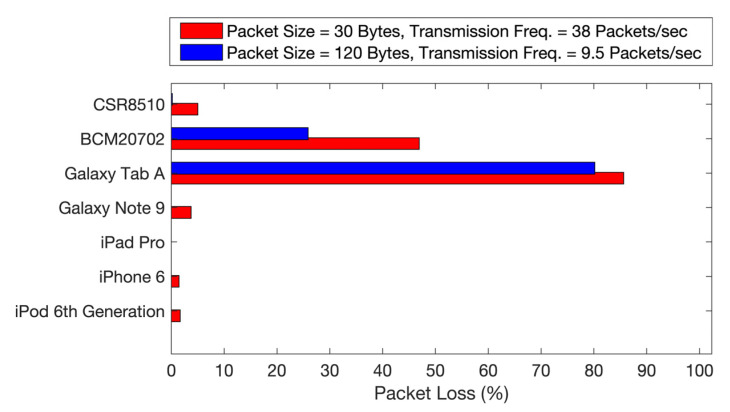
Evaluation and comparison of packet loss under reduced transmission frequency and data bundling.

These two observations were further confirmed by the test results shown in Figure 6. We compared the packet loss for the same final throughput under two different sets of parameters. The common MTU among the hosts picked in Table 1 is 120 bytes. Assuming that 120 bytes is the maximum packet size that can be safely transmitted to the hosts, it can be seen that packets of size 30 bytes and a transmission frequency of 38 packets/sec (CI = 105 ms, 4 packets per CI) can be reconfigured to avoid losses by bundling 4 packets together in a payload of 120 bytes using a transmission frequency of 9.5 packets/sec (CI = 105 ms, 1 packet per CI). The results in Figure 6 show that the packet losses in different hosts have been drastically reduced, with most hosts reporting zero loss when increasing the packet size and reducing the transmission frequency.

Furthermore, the packet loss under different power modes of the hosts was also tested. Smartphones like iPhone 6 provide an option to switch the device into “Low Power Mode”. Galaxy Note 9 provides various battery-saving modes like “High Performance”, “Optimized”, “Medium Power Saving”, and “Maximum Power Saving”. When the packet size and the transmission frequency were set to be 120 bytes and 9.5 Hz, respectively, the power mode of the host did not affect the packet loss rate, which remained at zero percent for all hosts tested.

### 3.2. Influence of External Environment on Data Loss

The distance and the objects between the host and peripheral determine the signal strength of the BLE connection perceived by the host. In most cases, the data transmission is initiated by the user from her/his smartphone (host), when the wearables (peripherals) are around. In this case, the distance between the host and peripheral is typically an arm’s length, e.g., within 1 m. However, when a hub like Raspberry Pi is used to initiate a periodic scan and transfer request, a larger distance is expected between the hub and peripherals; thus, the influence of RSSI and throughput on the reliability of data transmission need to be carefully evaluated.

With the ART, the user would synchronize their data at the end of the day to check the daily pollutant exposure. Meanwhile, with the WMD, the user would access the data immediately through the host right after the measurement is finished. Both scenarios most likely happen in a typical living room/bedroom or office environment. The average size of a regular living room or bedroom of a house in the USA is about 40 sq. meters [45].

**Figure 7:**
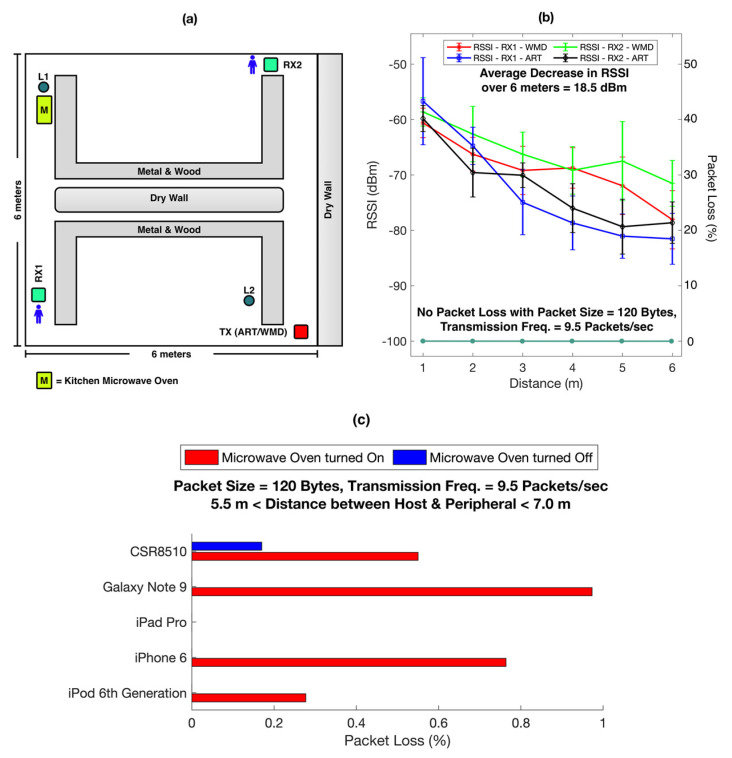
Evaluation of the influence of the external environment on packet losses in BLE transmission. (**a**) The room environment used to check the RSSI of peripherals. (**b**) Comparison of RSSI between peripheral and host at various distances across the room. (**c**) Packet loss due to the influence of microwave oven on BLE transmission.

As shown in Figure 7a, an environment representing a living room or office was chosen to evaluate the RSSI of the peripherals. The room had a metal shelf, wood desk, plastic chair, and drywall, all of which could serve as obstacles for the BLE data transmission. The environment also had 2.4 GHz and 5 GHz Wi-Fi network signals that provided internet connectivity to the hosts. Previous work showed a minimal impact of interference from these sources on BLE transmission [46,47]. Other miscellaneous power lines and live ethernet cables were present around these devices. The peripherals were placed at a fixed position labeled as “TX” at one corner of the room, while the host moved away from “TX” to approach the positions labeled “RX1” and “RX2”, during which the RSSI was measured at increasing distances. 

Figure 7b shows the relationship between signal strength of the peripherals (Primary Y-Axis) and the distance with the host (X-Axis). The host was moved up to 6 m from the peripheral, towards two opposite corners of the room. The signal strength decreased by an average of 18.5 dBm which is consistent with previous findings in an environment with obstacles such as wood, human body, metal, and brick wall [48]. The pattern of decrease in RSSI was similar between the peripherals, WMD, and ART. It is reported that an increase in distance between the host and the peripheral by 6 m has a negligible impact on the throughput of data transmission and energy consumption of the BLE 4 module [48,49]. Figure 7b also shows that no packet losses (Secondary Y-Axis) were experienced with the increasing distance between the host and peripheral (X-Axis).

A common household or work environment also includes appliances, such as a benchtop microwave oven, that emit radio signals in the same frequency range as that of BLE, which could potentially interfere with the peripheral’s signal [21]. We investigated the influence of the microwave oven on the data loss in BLE transmission.

As shown in Figure 7a, a kitchen microwave oven (Emerson MW8987B, rated 900W, full microwave power) was located at position L1. The oven heated a 300 mL cup of tap water at the highest power setting throughout the entire data transfer session. Another location at L2 (~6 m away from L1) was chosen based on a normal usage scenario, for testing the influence of the microwave oven on packet transmission over BLE. 

We observed that if both the peripheral and the host were located together (within 0.5 m) at L1 or L2, no packet losses were observed under a packet size of 120 bytes and a transmission frequency of 9.5 Hz. However, when one of them was located at L1 and the other at L2, a consistent packet loss pattern was observed when the microwave was turned on, as shown in Figure 7c. In this case, the hosts showed a packet loss of less than one percent. Though this packet loss is not significant, it is critical when high data transmission reliability is required in certain application scenarios.

### 3.3. Mitigation Protocol against Packet Loss over Bluetooth Low Energy

As discussed before, the packet loss rate varies greatly across various host devices, and it also depends on the internal parameter settings and the external environment under which the hosts operate. Even at a low transmission frequency and data bundling, it has been observed that the external environment can affect the data transmission in BLE. Therefore, mitigation strategies are needed to avoid packet loss in BLE for the wearable healthcare ecosystem.

**Figure 8:**
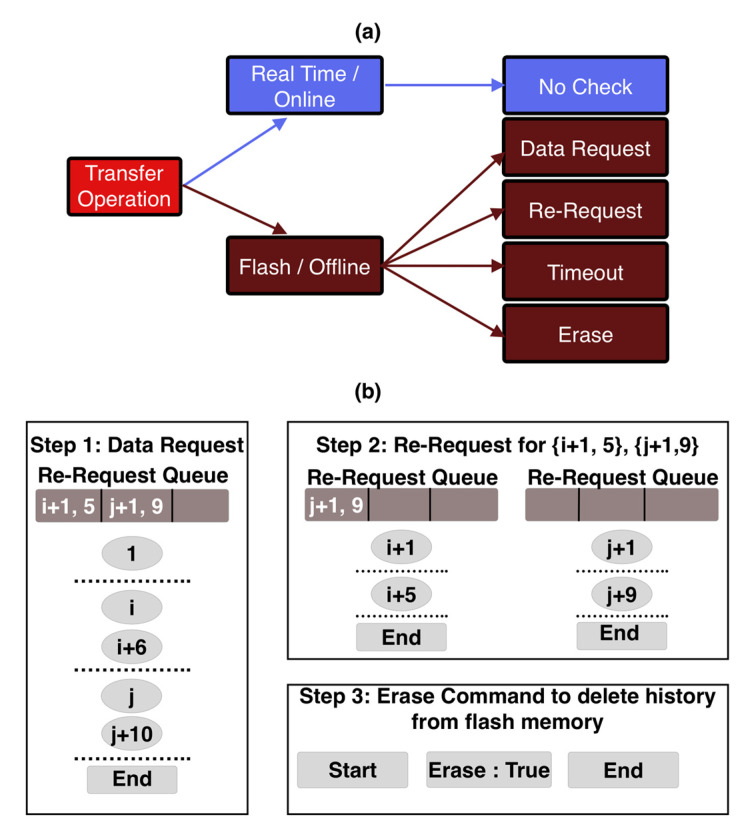
Data transmission protocol for BLE to mitigate packet loss. (**a**) Two transmission modes between the peripheral and the host. (**b**) Queue based mechanism to track packet loss and re-request packet before ending the session.

The peripheral can opt to work under one of the two modes, real-time mode or offline mode, as illustrated in Figure 8a. In the real-time mode, data from the peripherals are transmitted to the host as soon as it is available. This host does not check for data loss and can end the transmission at will. WMD adopts this model since the user needs to actively interact with the device in real-time to follow the measurement procedures and access the test results.

**Figure 9:**
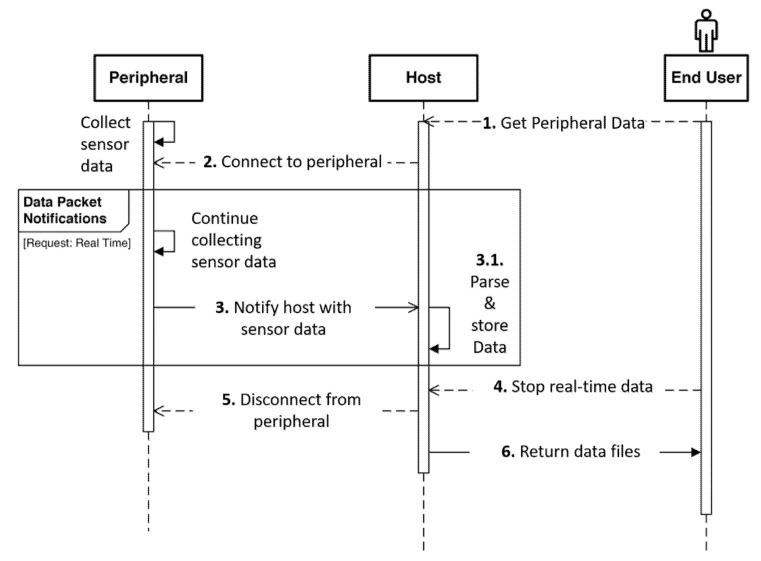
Sequence diagram indicating the series of interactions involved with data requested in real-time mode.

To avoid data losses in real-time mode, the peripheral needs to reduce its transmission frequency. The WMD computes respiratory information like respiratory rate, tidal volume, and minute ventilation data, and accumulates this data over time. The accumulated values are transmitted over BLE at 1 Hz. The host receives the data packets and processes the data using the timestamps of the data. Since respiratory parameters change much slower than 1 Hz, a transmission frequency of 1 Hz is sufficient for real-time data transmission between the peripheral and the host. Typically, the respiration-related signals are processed over a given time window to improve the signal-to-noise ratio. The host keeps receiving data packets and processing data in the time window. Any occasional packet loss within this time window can be mitigated by using the upcoming packet and averaging the data over a slightly larger window. Since the sampling rate of the wearable is much faster than the actual change of physiological parameters, our results show that this mitigation method is still effective to ensure the accuracy of the measurement. The sequence of operations and interactions between the peripheral and the host in real-time are illustrated in Figure 9. It starts with the end user initiating a request by interacting with the application on the host. The host connects to the peripheral and sends out a command packet for real-time data. The peripheral continues to collect the sensor data and notifies the user with new data packets generated using the latest sensor information. The host application may choose to display necessary packet information to the end user. The mode ends when the end user exits the application.

In the offline mode, the peripheral is not expected to maintain a constant pairing with the host. In this mode, the peripheral is not required to be constantly available within the BLE range of the host. Devices like ART monitor environmental exposure throughout the day continuously and passively without any need for user interaction. Hence, these peripherals store all measured data in their flash memory. After the daily monitoring, the user can initiate a request for the sensor data transmission on the host and the peripheral can transmit all the collected data to the host at once. The offline mode is also more energy-efficient since constant communication between the peripheral and the host is not maintained, which is helpful to extend the operation time of the battery in the peripheral device.

**Figure 10:**
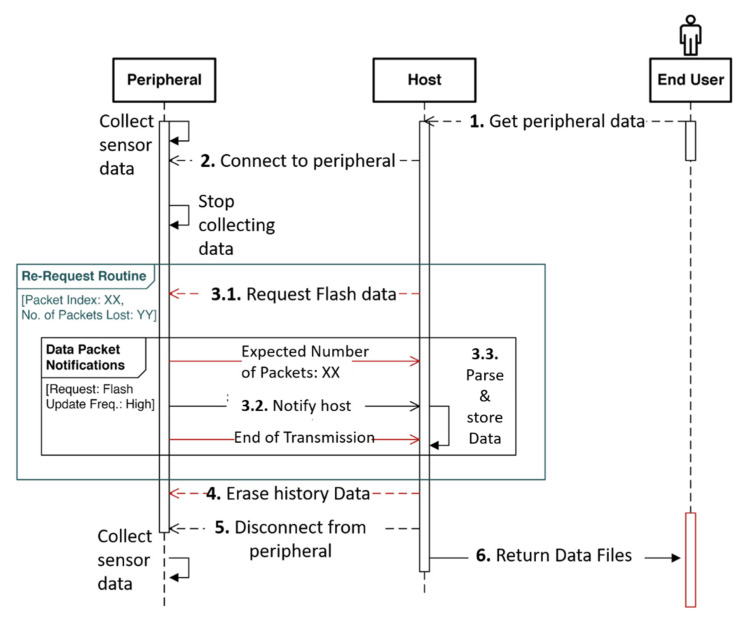
Sequence diagram indicating the series of interactions involved with requests for transmitting data that has been stored in the flash memory in the peripheral. The end user initiates a request to get stored information from the peripheral. The host application connects to the target peripheral and sends out a command packet and the peripheral transmits the flash packets. After the end of initial transmission, the host verifies the sequence of indices received and invokes a re-request routine for any lost packets. The session ends with the host terminating the connection by sending out a command to erase the existing flash data on the peripheral.

As illustrated in Figure 8b, the ART peripheral transmits all data stored in its flash memory to the host. The host keeps track of the missing packet indices between the incoming packets in a queue structure. When the host receives the End Packet from the peripheral, it checks for missing data in the queue and initiates a re-request using the request command packet. However, this time it mentions the specific packet indices that need to be transmitted again. The host keeps track of any missing indices in the re-request routines and adds them to the queue for subsequent re-requests. This continues until the host receives all the expected packet indices and finally ends the transmission session by sending the erase command. When the peripheral receives the erase command, it erases the flash memory to make space for future sensor data storage. This transmission protocol ensures that none of the data gets lost in the offline mode. The sequence of operations and interactions are illustrated in Figure 10.

## 4. Discussion

A BLE-based wearable healthcare ecosystem was created using two homemade peripherals and various commercially available hosts to evaluate packet loss over BLE transmission and develop a mitigation protocol. The performance of BLE-based data transmission was evaluated under different connectivity settings and the influence of the external environment on data loss was investigated. Based on the test results, we suggested a reliable and generic BLE transmission protocol that can be easily adapted across different iOS and Android hosts. The proposed protocol provides flexibility to the peripheral device to work with the host either in real-time mode for timely data transmission or in offline mode for bulk transmission when requested by the host. The mitigation protocol developed in this research can also be widely applied to various other BLE-based ecosystems such as body sensor networks (BSN), the Internet of Things (IoT), and smart homes. 

Though this research has studied the possible interference in a typical home and office setting, there is still a need to investigate other sources. With the growing daily use of Wi-Fi and other wireless communication techniques, it is important to explore ways and means to reduce their impact on BLE. A comprehensive evaluation is necessary to understand packet loss in harsh environments like smart cities or IVWSN which are also conditions a user might encounter.

For the mitigation protocol, a detailed study is required in the future to investigate its performance. We envision that this protocol can be made more dynamic by exchanging the MTU size and maximizing the bundling on the peripheral side to enhance the overall user experience and achieve the best lossless throughput. Furthermore, the peripheral can be designed to exchange the packet structure with the host before the packet transmission starts, enabling the addition of more peripheral devices to the BLE-based wearable healthcare ecosystem for more comprehensive sensing and monitoring.

## Figures and Tables

**Table 1:** Specifications of the host and peripheral devices.

Device	Operating System (OS)	BLE Chipset
iPod Touch 6th Gen	iOS 12.0.1	BCM4335
iPhone 6	iOS 12.4.8	BCM4345
iPad Pro	iOS 13.6	----
Samsung Note 9	Android 10	SDM845
Galaxy Tab A	Android 5.1.1 (Lollipop)	BCM4330
Raspberry Pi 3 B+	Raspberry Pi OS	BCM20702
Raspberry Pi 3 B+	Raspberry Pi OS	CSR8510
ART	TI-RTOS	CC2640
WMD	TI-RTOS	CC2640
